# Serum response factor targets Cyr61 to facilitate chronic progression after ischemic acute kidney injury through renal tubular epithelial–myofibroblast transdifferentiation

**DOI:** 10.1186/s40001-025-03716-8

**Published:** 2025-12-24

**Authors:** Lin Che, Lingyu Xu, Chenyu Li, Chen Guan, Quandong Bu, Congjuan Luo, Hong Luan, Bin Zhou, Chengyu Yang, Xiaofei Man, Hui Zhang, Yan Xu, Long Zhao

**Affiliations:** Department of Nephrology, The Affiliated Hospital of Qingdao University, 16 Jiangsu Road, Qingdao, 266003 China

**Keywords:** Serum response factor, Cysteine-rich protein 61, Epithelial–myofibroblast transdifferentiation, Ischemia/reperfusion, Acute kidney injury

## Abstract

**Objectives:**

To explore the regulation and function of serum response factor (SRF)/cysteine-rich protein 61 (Cyr61) pathway in renal tubular epithelial–myofibroblast transdifferentiation (EMyT) in the chronic progression after ischemic acute kidney injury (AKI).

**Methods:**

The expression of SRF, Cyr61, myofibroblast markers (collagen-3, α-SMA and vimentin) and epithelial markers (E-cadherin and ZO-1) were examined in mouse renal tubular epithelial cells (TCMK-1 cells) under hypoxia/reoxygenation (H/R) treatment or rat renal medulla tissue samples after ischemia/reperfusion (I/R) treatment. SRF was overexpressed by pcDNA–SRF plasmid and suppressed by CCG-1423 (a small molecule inhibitor of SRF) or SRF siRNA to study how SRF influences renal tubular EMyT through Cyr61 in the chronic progression after AKI.

**Results:**

In TCMK-1 cells under H/R treatment and renal medulla tissue from I/R rats, the SRF along with Cyr61, collagen-3, α-SMA and vimentin expression was upregulated, while E-cadherin and ZO-1 expression was downregulated. SRF upregulation in TCMK-1 cells increased Cyr61 expression. Blockade of SRF by an SRF-specific siRNA or CCG-1423 reduced Cyr61 induction, protected renal tubular epithelial cells from undergoing EMyT and improved the chronic progression after ischemic AKI both in vitro and in vivo.

**Conclusions:**

Increased SRF/Cyr61 pathway activity promotes EMyT and dysfunction in renal tubular epithelial cells in the chronic progression after AKI. Targeting SRF with CCG-1423 may be an attractive therapeutic strategy in the chronic progression after AKI.

## Background

Acute kidney injury (AKI) as a common critical syndrome have high incidence and mortality but with low diagnosis rate [1]. It initiates swiftly, its progression is rapid, the state of the condition is critical, with average mortality rate ranges from 50% to 65.2%. Approximately 35.8% to 58% of survivors transition to chronic kidney disease (CKD), while 7.5% of them require maintenance dialysis. [2]. Epidemiological studies have documented a worldwide occurrence of AKI spanning from 1 to 66%. Presently, China accommodates 2.9 million individuals diagnosed with AKI, leading to a substantial treatment expense of up to 13 billion US dollars. This imposes a significant strain on the healthcare system and its resources [3, 4]. In a clinical context, kidney ischemia–reperfusion (I/R) injury stands as a frequent etiology of AKI, exerting a considerable impact on both the initial functional recuperation and the extended viability of grafts post kidney transplantation [5]. Instances of kidney I/R injury frequently manifest subsequent to infection, trauma, or surgical procedures, and there remains a scarcity of viable interventions capable of mitigating symptoms and enhancing patient survival [6]. Pathophysiologically, renal I/R injury tends to exhibit traits, such as tubular epithelial–myofibroblast transdifferentiation (EMyT), inflammation, and compromised vascular functionality, often accompanied by pronounced cellular demise [6].

Renal tubule epithelial cells (RTECs) constitute the primary cellular component within the kidney and are vulnerable to various forms of damage, e.g., toxins, hypoxia, mechanical stress, and senescence [7]. Research has demonstrated that the loss of RTEC polarity and the occurrence of EMyT represent early distinctive alterations that precipitate renal tubulointerstitial fibrosis following kidney injury. These changes also hold a pivotal role in the advancement of AKI toward the development of CKD [7–9]. EMyT represents a phenotypic transition mechanism, whereby epithelial cells undergo a conversion into myofibroblast-like cells, instigated by a specific signaling pathway. This process is characterized by a reduction in epithelial markers, such as zonula occludens 1 (ZO-1) and E-cadherin, accompanied by over-activation in cytokeratin, vimentin, α-smooth muscle actin (α-SMA), fibroblast-specific protein-1 (FSP-1), collagen-3, and fibronectin (FN), all of which experience elevated expression levels[10].

Transcription factors are considered key regulators of gene transcription, and they have important implications in the progression of human diseases. Serum response factor (SRF) is a highly conserved cis-acting factor belonging to the MADS-box transcription factor family [11]. The DNA site that SRF binds to is the serum response element (SRE), which controls the expression of cytoskeletal proteins and contractile proteins and promotes cell migration and differentiation [12]. SRF is expressed in a variety of cells and is not histiocytic specific, with more than 300 genes that can bind to it. SRF acts with cofactors to activate immediate early genes such as c-fos and Cyr61 in response to specific stimuli [13]. A recent study showed that in the process of renal fibrosis, SRF promoted the secretion of connective tissue growth factor (CTGF) and led to RTECs EMyt [14]. It was also reported that SRF had significant roles in progressive carcinoma, specifically in the process of EMyT and in the ability of migration and invasion in many cancers, for example, breast cancer [15], melanoma [16], renal cell carcinoma (RCC) [17]. However, how SRF regulates renal tubular injury in I/R AKI remains mostly unknown.

Herein, in this study, it is investigated whether SRF played a role in mouse RTECs under hypoxia/reoxygenation (H/R) treatment and I/R rat model. In addition, it is also evaluated whether there is a therapeutic potential of SRF/Cyr61 pathway in the chronic progression after AKI.

## Methods

### Materials

Anti-β-actin (1:1000, BS1002) and anti-pSRF (1:1000, BS4177) antibodies were obtained from Bioworld Technology (Louis Park, MN, USA); anti-SRF antibody (1:500, sc-335) were obtained from Santa Cruz (Santa Cruz, CA, USA); and anti-collagen-3 antibody (1:1000, ab184993), anti-E-cadherin (1:1000, ab76055), anti-Cyr61 (1:1000,ab230947), anti-α-smooth muscle actin (α-SMA) (1:1000, ab5694) and anti-vimentin (1:1000, ab92547) antibodies were obtained from Abcam (Cambridge, MA, USA). Anti-collagen Iα1 were obtained from Proteintech (Wuhan, Hunan, China). CCG-1423 was obtained from Cayman Chemical (Ann Arbor, MI, USA).

### Animal protocols

Clean, healthy male SD rats were purchased from Jinan Yuepeng Animal Center, weighing approximately 150–180 g. 40 SD rats were adaptively fed for 1 week and then randomly divided into the following 3 groups: (I) Sham group(n = 6); (II) I/R group (n = 6); and (III) I/R rats treated with CCG-1423 diluted in a 1:100 solution of dimethyl sulfoxide (DMSO): phosphate-buffered saline (PBS) (I/R + CCG-1423 group n = 6). CCG-1423 was administered by daily intraperitoneal injection at doses of 0.02 mg/kg of BW for 4 weeks beginning on the day after operation. As a control, the same volume of the vehicle (100 μL/100 g of BW) was administered to the sham and I/R group animals. After being anesthetized, the right kidneys of all experimental rats were removed, and the left renal pedicles were closed with non-invasive vascular clamp for 40 min and then unclamped. During this process, we observed that the kidney color gradually changed from bright red to dark red, and then rapidly from dark red to bright red after unclamped. The left kidney of the control group was not clipped. We collected 2 ml of blood from the rat hearts and removed the left kidneys at 4 weeks after the operation. The levels of serum creatinine (Scr) and blood urea nitrogen (BUN) were detected by Olympus AU2700 automatic biochemistry analyzer. All animal experiments complied with the ARRIVE guidelines. All experiments were performed in accordance with the Chinese guidelines on the use and care of laboratory animals and were approved by the Laboratory Animal Welfare and Ethics Committee of the Affiliated Hospital of Qingdao University.

### Sample collection, histopathological analysis and immunohistochemical staining

Kidney in paraffin sections were deparaffinized and hydrated, then fixed with 4% paraformaldehyde and immunohistochemically stained as previously described [18]. The secondary antibodies used were: Horse Anti-Mouse IgG Polymer Kit (Peroxidase) (Vector Laboratories, Catalog #: MP-7402); Horse Anti-Rabbit IgG Polymer Kit (Peroxidase) (Vector Laboratories, Catalog #: MP-7401). The Images were visualized with an Olympus inverted microscope IX71 with CellSens software (Olympus, Melville, NY, USA). Quantitative evaluation of collagen and immunohistochemical staining was performed by computerized analysis using Image J (NIH).

### Cell culture and treatment

TCMK-1 cells were obtained from the American Type Culture Collection (BNCC, Beijing, China). Cells were cultivated at 37 °C in DMEM (HyClone, Logan, UT, USA) supplemented with 10% fetal calf serum (Gibco, Langley, OK, USA) and 1% penicillin/streptomycin (Gibco, Langley, OK, USA). CCG-1423 was dissolved in DMSO and administrated at the dose of 2 μM). At the same time, the same volume of DMSO was given to the control group. H/R group cells were exposed to 12 h of hypoxia (5% CO2, 1% O2, and 94% N2) followed by 6 h, 12 h, 24 h, 48 h of reoxygenation for the experimental time period, respectively. Control cells were incubated under normoxic conditions without a medium change. Each experiment was repeated independently three times.

### Small-interfering RNA (siRNA) and a luciferase assay

An SRF-specific siRNA (5’-GACCTGCCTCAACTCGCCAGAC-3’) was described previously [19]. Cyr61 upstream regions relative to transcription start site were cloned into pGL3-Basic vector (Promega, Madison, Wisconsin, USA), upstream of firefly luciferase coding sequence via PCR and subsequent ligation. TCMK-1 cells were transfected with the pcDNA3.1 or pcDNA–SRF plasmid and a nonspecific siRNA or the SRF-specific siRNA along with empty vector or pGL3–Cyr61 plasmids using HiPerFect Transfection Reagent (Qiagen, Duesseldorf, Germany). Luciferase activities were evaluated through a luciferase assay system (Promega, Madison, Wisconsin, USA), and the luminescence was measured using an illuminometer (BMG FLUOstar OPTIMA, Germany).

### Plasmids and transfection and quantitative RT-PCR

These methods were described previously [20]. All the primer sequences are reported in Table 1.

**Table 1:** Primers used in this study

Gene	Forward (5’ → 3’)	Reverse (5’ → 3’)
SRF	GCACAGACCTCACGCAGA	ATGTGGCCACCCACAGTT
ZO-1	GGAAACCCGAAACTGATGCTATGG	AACTGGCTGGCTGTACTGTGAG
Fibronectin	AGACCCCAGGCACCTATCAC	TCGGTCACTTCCACAAACTG
Cyr61	GCAGTTGGAAAAGGCAGCTC	ACAGGTCTTTGAGCACTGGG
GAPDH	GGATTTGGTCGTATTGGG	GATGATCTTGAGGCTGTTGTC

### Western blot

Proteins (30 μg/lane) were separated by SDS–PAGE and western blotting was performed as previously described. The secondary antibodies used were: goat–anti-rabbit IgG (H + L) (1:5000; Invitrogen, Catalog # A21109) and goat–anti-mouse IgG (H + L) (1:5000; Invitrogen, Catalog # A21203). Protein bands were scanned and quantified using the Li-cor Odyssey infrared scanning system (Li-COR Biosciences, Lincoin, Nebraska). Quantitative evaluation was performed by computerized analysis using Image J (NIH).

### Immunofluorescence staining

TCMK-1 cells were fixed for 20 min at room temperature in 4% paraformaldehyde and immunofluorescence staining was performed as previously described [20]. The secondary antibodies used were: FITC-conjugated goat–anti-rabbit IgG (H + L) (ZSGB-BIO, Catalog # ZF-0311); FITC-conjugated goat–anti-mouse IgG (H + L) (ZSGB-BIO, Catalog # ZF-0312). The slides were viewed under Nikon epifluorescence microscope.

### Transwell chamber migration assay

The motility of TCMK-1 cells was determined using a transwell chamber migration assay, as previously described [20].

### Molecular docking

For the molecular docking AutoDock (version 4.2.6) was used. The PDB format files of hesperetin was initially obtained from the NCBI PubChem Compound database (https://www.ncbi.nlm.nih.gov/pccompound/) in SDF format and then converted using Open Babel GUI (version 2.4.1). The PDB format files of proteins were downloaded from the Uniprot (https://www.uniprot.org/). The files of hub gene-encoded proteins, set as receptors, were prepared after removing solvent molecules and ligands, and applying operations, such as hydrogenation and electron handling. Subsequently, a PDBQT format file of hesperetin, set as the ligand, was generated for molecular docking analysis. Results were analyzed using AutoDockTools (version 1.5.7), and visual simulation was performed using PyMOL (version 2.4.1).

### Bioinformatics:

*Bulk RNA-seq:* GSE76882 and GSE87024 raw data were downloaded from the GEO database. The raw data underwent quality control and normalization using a robust multi-array averaging algorithm. Differential expression analysis was conducted by the Linear Models for Microarray Analysis (Limma) package [21]. The *p* value was adjusted using the Benjamini and Hochberg method. Differentially expressed genes (DEGs) were identified based on an absolute log2FC > 2 and an adjusted *p* value (adj. P) < 0.05.

*Single-cell RNA-seq (scRNA-seq):* The raw data or count matrices were obtained from GSE139107 [22]. The scRNA-seq data were processed using the Scanpy [23] pipeline, which facilitated the integration and merging of different batches of data. Quality control was performed by utilizing the threshold values as reported in the original article. The normalization of the data was conducted using the scran [24] package, which included assuming equal size factors, normalizing the library size to counts per million, and log-transforming the count data. The 3000 most variable genes were used for the principal component analysis. To eliminate technical differences and preserve biological differences, the harmony integration pipeline was employed, which reduces data dimensions and removes batch effects [25]. The reduction of data dimensions and Uniform Manifold Approximation and Projection (UMAP) was applied for unsupervised clustering based on the first 50 integrated principal components. The resolution of clustering was determined accordingly. The gene enrichment score was evaluated by "scanpy.tl.score_genes()" functions [26].

### Statistical analysis

All data are shown as the mean ± SEM. Every experiment was repeated at least 3 times. Statistical analysis was performed using SPSS (version 20.0; SPSS Inc., Chicago, IL, USA). Intergroup comparisons were conducted through one-way analysis of variance or an unpaired *t* test. *P* < 0.05 was considered statistically significant.

## Results

### SRF and Cyr61 overexpressed in AKI patients’ kidney

**Fig. 1:**
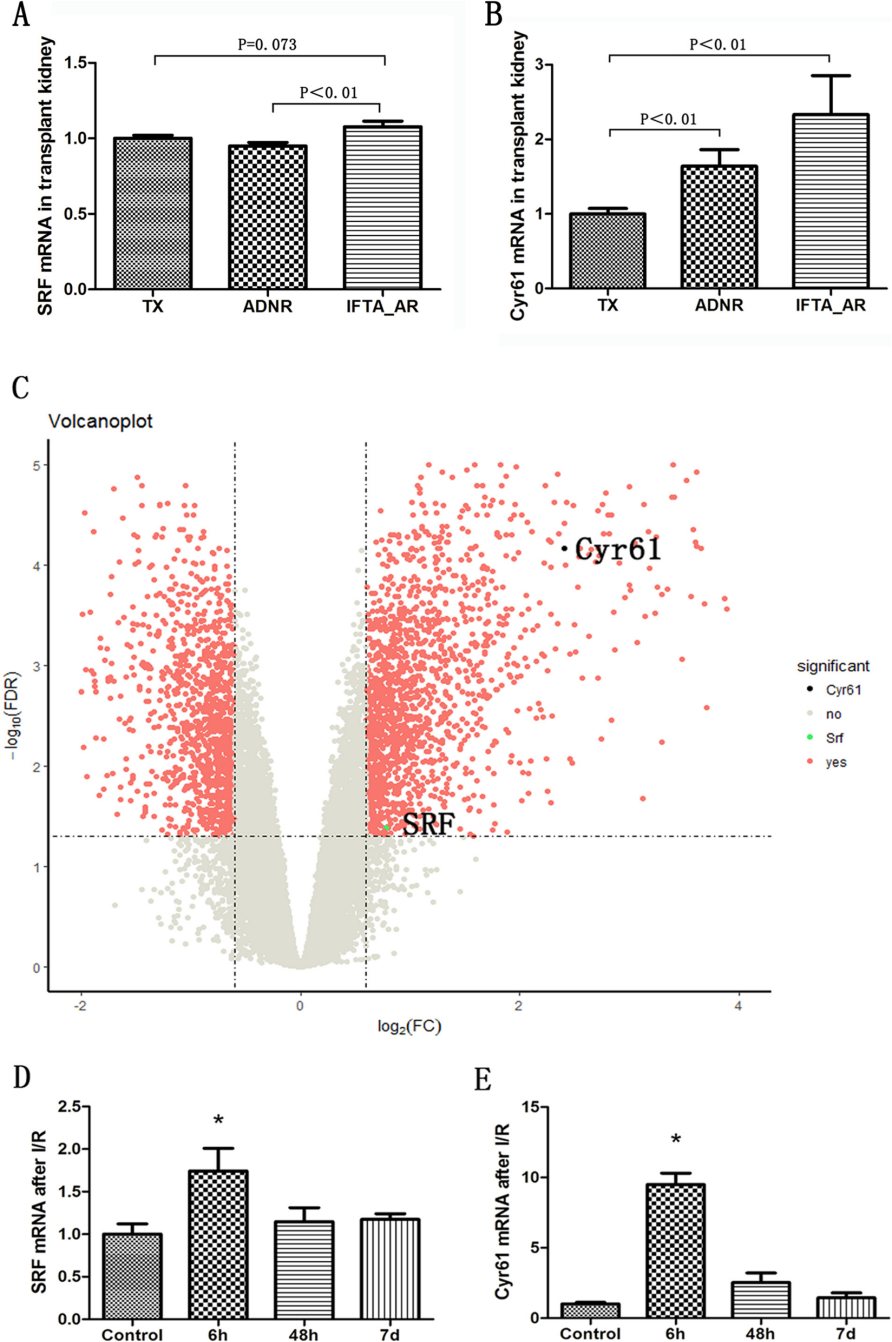
Expression of SRF and Cyr61 genes was increased in the kidney of AKI patients. **A**, **B** Expression of SRF and Cyr61 genes in transplanted kidneys with acute rejection. **C** Volcanic maps showing transcriptome changes 6 h after I/R renal injury. The ordinate represents the logarithmic gene expression multiple, and the ordinate represents the logarithmic corrected *p* value. The vertical dashed line is the gene up-regulated by a factor of 2 or down-regulated by a factor of 0.5, and the horizontal dashed line is the corrected *p* value equal to 0.05. **D**, **E** Expression of SRF and Cyr61 genes in kidney of patients with ischemic AKI at different timepoints, *P < 0.05 compared with control group. *TX* normal function kidney transplantation, *ADNR* acute renal failure without rejection, *IFTA_AR* acute rejection with interstitial fibrosis and tubular atrophy

By utilizing the mRNA chip data set from human kidney transplantation (GSE76882), we conducted an analysis on SRF and Cyr61 expressions. Our findings revealed a significant increase in the mRNA expression of both within transplanted kidneys experiencing acute rejection, accompanied by interstitial fibrosis and renal tubule atrophy. Notably, Cyr61 exhibited a more pronounced increase (Fig. 1A, B). Moreover, the expression of SRF and Cyr61 mRNA in the kidneys of patients with ischemic AKI (GSE87024), which confirmed that SRF and Cyr61 mRNA were simultaneously expressed in AKI, with Cyr61 displaying a more noticeable alteration (Fig. 1C–E). These results collectively indicate an elevation of SRF and Cyr61 in AKI stemming from diverse causes. Given the more pronounced changes in Cyr61, it is suggested that SRF may exert its functions on AKI through Cyr61.

### SRF and Cyr61 expressed differently in time- and cell-specific manner post-AKI

**Fig. 2:**
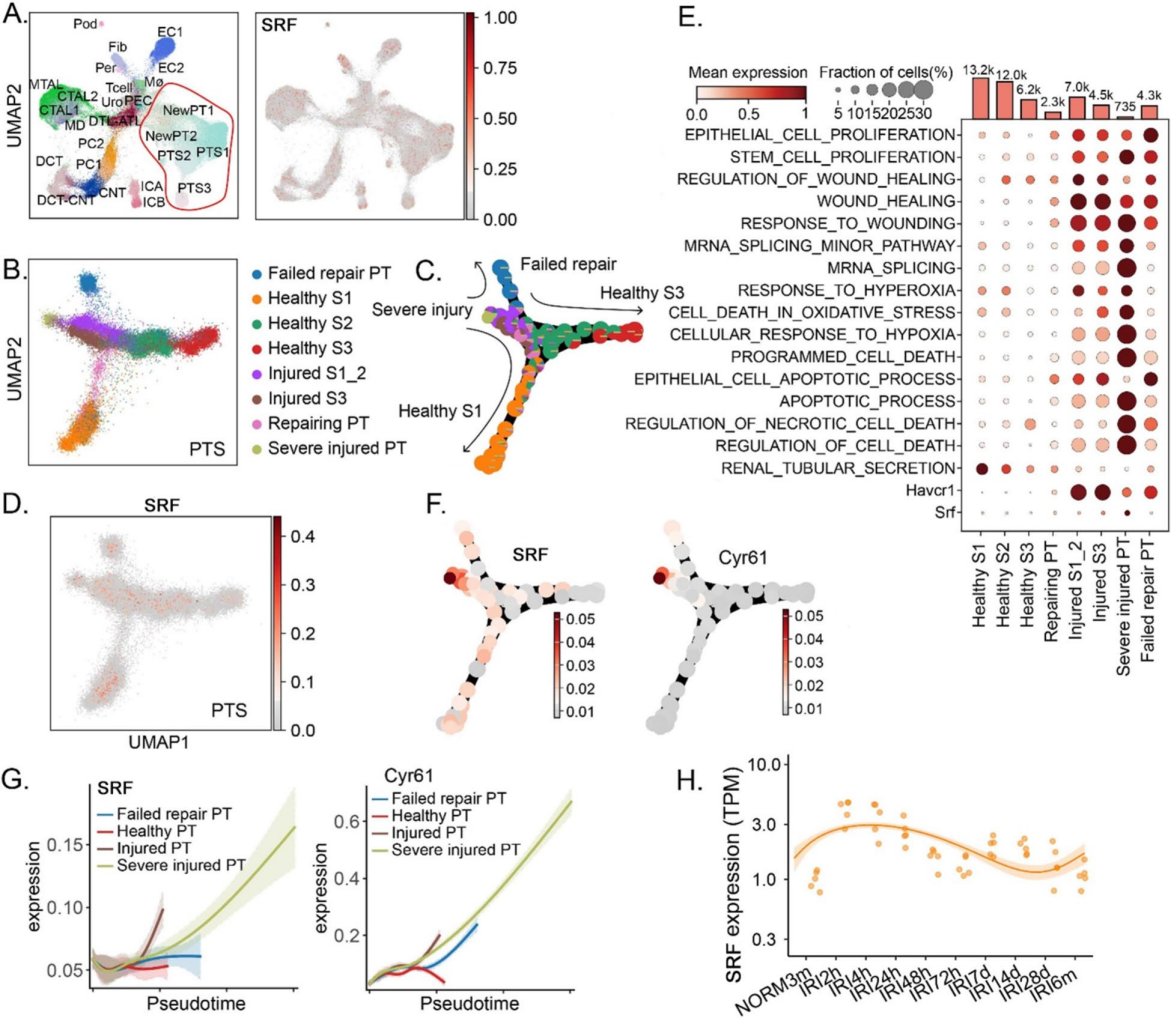
Expression of SRF and Cyr61 genes at different times and in different cell types after ischemic AKI was analyzed using single-cell sequencing data from a public database. **A** UMAP visualization of kidney single-cell transcriptomes showing distinct cell clusters and the expression of SRF. **B** Subclustering of proximal tubular segments identifies distinct cellular states, including healthy S1–S3 segments, injured S1/S3 cells, repairing PT, severely injured PT, and failed-repair PT populations. **C** Pseudotime trajectory analysis of PT cells demonstrates the progression from healthy to injured, repairing, and failed-repair states. **D** UMAP plots showing the expression of SRF in proximal tubule subclusters. **E** Dot plot summarizing pathway enrichment and representative marker expression across PT subpopulations. **F** Pseudotime trajectory mapping illustrates the dynamic induction of SRF and Cyr61 during the transition from injury to failed repair. **G** Expression kinetics of SRF and Cyr61 along pseudotime demonstrate early activation of injury markers followed by progressive upregulation of SRF and Cyr61 in severely injured and failed-repair PT populations. **H** Temporal changes in SRF expression in kidneys after I/R injury. *CNT* connecting tubule, *CTAL* thick ascending limb of loop of Henle in cortex, *DCT* distal convoluted tubule, *DTL* descending limb of loop of Henle, *EC* endothelial cells, *Fib* fibroblasts, *ICA* type A intercalated cells of collecting duct, *MTAL* thick ascending limb of loop of Henle in medulla, *Mø* macrophages, *PT* proximal tubule, *PC* principle cells of collecting duct, *PT-S1* S1 segment of proximal tubule, *PT-S2* S2 segment of proximal tubule, *PT-S3* S3 segment of proximal tubule, *Pod* podocytes, *Uro* urothelium

We characterized the expression of SRF and Cyr61 in AKI using the GSE139107 single-cell sequencing data set of kidney cell after renal ischemia–reperfusion injury (Fig. 2A). The expressions of SRF and Cyr61 gradually increased in renal tubular epithelial cells (Fig. 2B–F) with the extension of the time after ischemic AKI. The SRF is correlated with the apoptotic and necrotic cell death and the cell proliferation gene enrichment (Fig. 2E). Both of the SRF and Cyr61 were very similar in terms of trends and highly expressed cell types (Fig. 2G), suggesting that SRF is likely to play a role in the progression of chronicity after ischemic AKI through Cyr61. The information is further confirmed in Fig. 2H, indicating that the expression of SRF is notably high in the kidney undergoing IRI, reaching its peak between 4 h and 28 days.

### H/R increased SRF expression and mediated EMyT of TCMK-1 cells

**Fig. 3:**
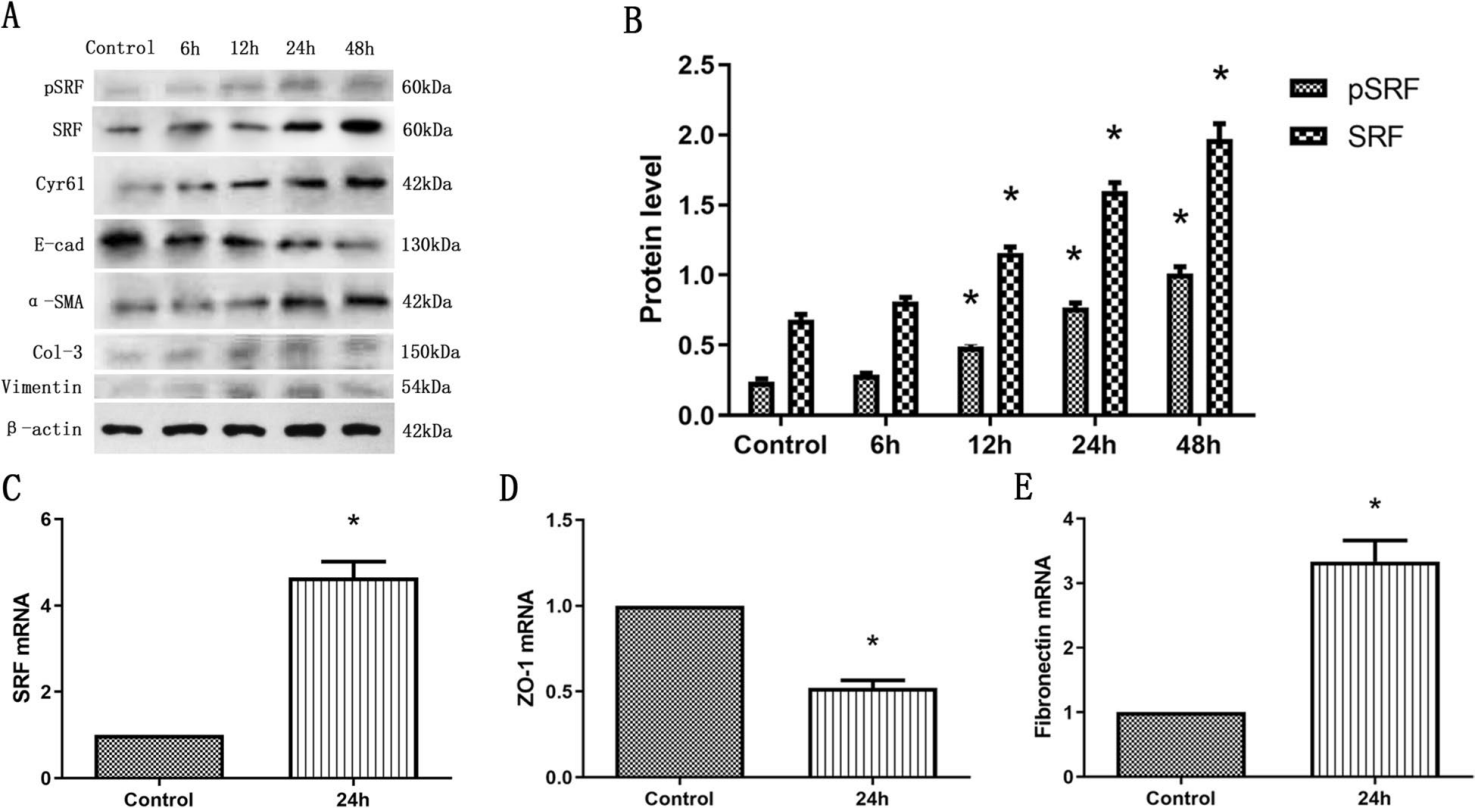
H/R increased SRF expression and mediated EMyT of TCMK-1 cells. **A** Western blot analysis showing that H/R mediated the upregulation of phosphorylated SRF (pSRF), SRF, collagen-3, α-smooth muscle actin (α-SMA), and vimentin expression and the downregulation of E-cadherin expression in a time-dependent manner. **B** Quantitative determination of relative SRF levels. **C–E** Quantitative RT–PCR analysis of SRF, ZO-1 and fibronectin (FN) mRNA expression. Data are representative of at least three independent experiments. *P < 0.05 versus the control group

Both the protein and mRNA expression of SRF were increased after H/R treatment by a time-dependent manner. What is more, the protein expression of phosphorylated SRF (pSRF) was also upregulated, which is taken for the biochemically activated form of SRF (Fig. 3A–C). In addition, the treated TCMK-1 cells tended to undergo EMyT. Western blot showed that H/R suppressed E-cadherin expression and induced collagen-3, vimentin and α-SMA upregulation by a time-dependent manner (Fig. 3A). Besides, as described in Fig. 3D, E, H/R suppressed the mRNA expression of ZO-1 and upregulated the mRNA expression of FN. Interestingly, the expression of Cyr61 was also upregulated in TCMK-1 cells after H/R stimulation, which indicates that Cyr61 might be correlated to SRF (Fig. 3A).

### SRF overexpression induced EMyT and migration capability of TCMK-1 cells

**Fig. 4:**
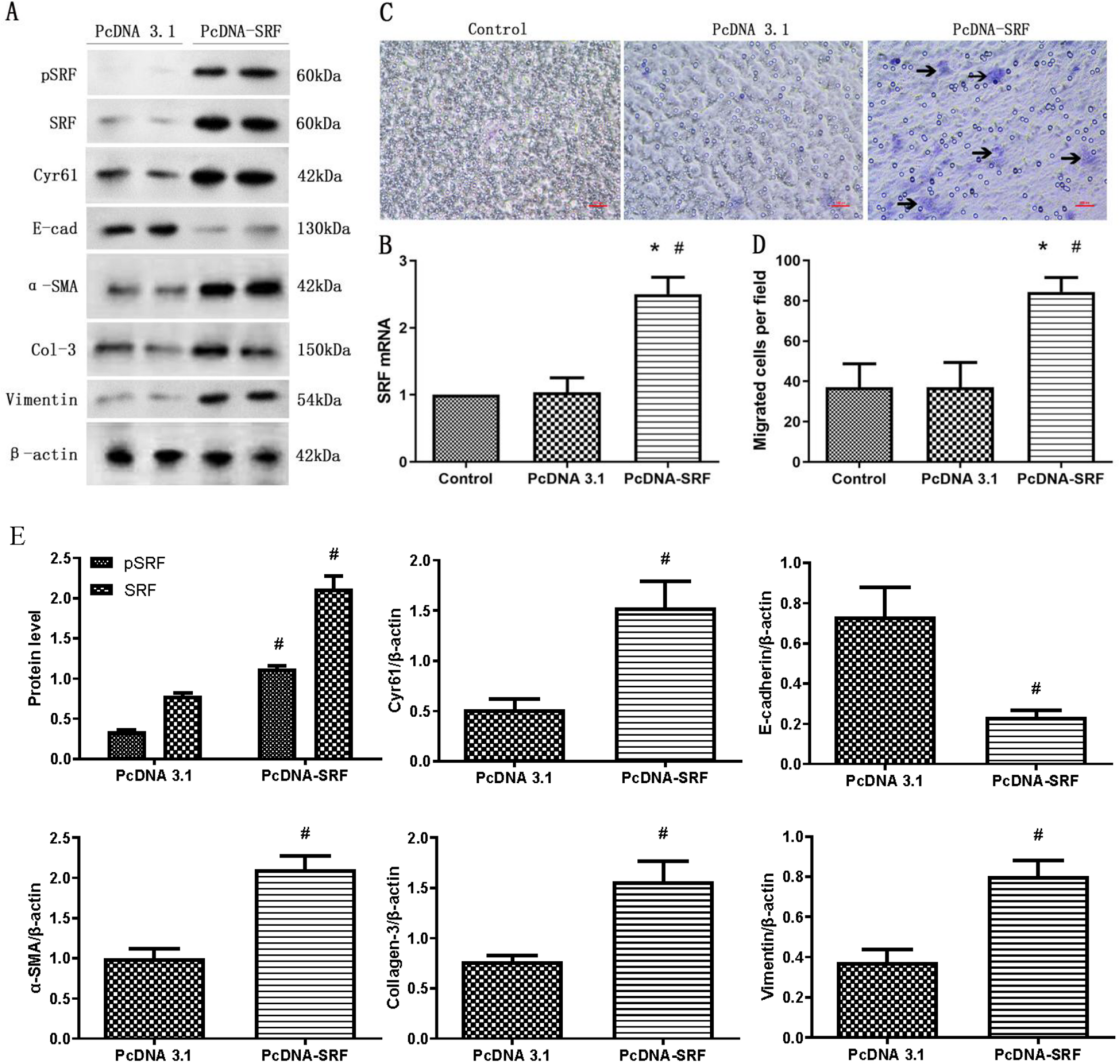
SRF overexpression induced EMyT and migration of TCMK-1 cells. **A** Western blot analysis showing the induction of pSRF, SRF, Cyr61, collagen-3, α-SMA, and vimentin expression and a reduction in E-cadherin expression. **B** Quantitative RT–PCR analysis of SRF mRNA expression. **C** Representative micrographs of the transwell chamber migration assay at an original magnification of 200 ×. Arrowheads indicate some of the migrated cells. Bar = 50 μm. **D** Quantitative analysis of the number of migrated TCMK-1 cells per field in three groups. **E** Quantitative determination of the pSRF, SRF, Cyr61, E-cadherin, α-SMA, collagen-3 and vimentin protein levels. Data are representative of at least three independent experiments. *P < 0.05 versus the control group; ^#^*P* < 0.05 versus the pcDNA3.1 group

As depicted in Fig. 4A, B and E, the introduction of pcDNA–SRF plasmids led to an elevated expression of both SRF mRNA and protein within TCMK-1 cells. As evidenced by the western blot analysis in Fig. 4A, E, the ectopic presence of SRF resulted in heightened levels of Cyr61, collagen-3, vimentin, and α-SMA, alongside a reduction in E-cadherin levels within TCMK-1 cells. These findings signify that the overexpression of SRF in TCMK-1 cells prompts a phenotypic shift reminiscent of EMyT, which may be related to Cyr61. Furthermore, the overexpression of SRF notably amplified the migratory capability of TCMK-1 cells across transwell filter membranes (Fig. 4C, D), indicating that the induction of EMyT solely through heightened SRF expression was adequate to enhance the migratory potential of TCMK-1 cells.

### Suppression of SRF preserved the phenotype of TCMK-1 cells post-H/R

**Fig. 5:**
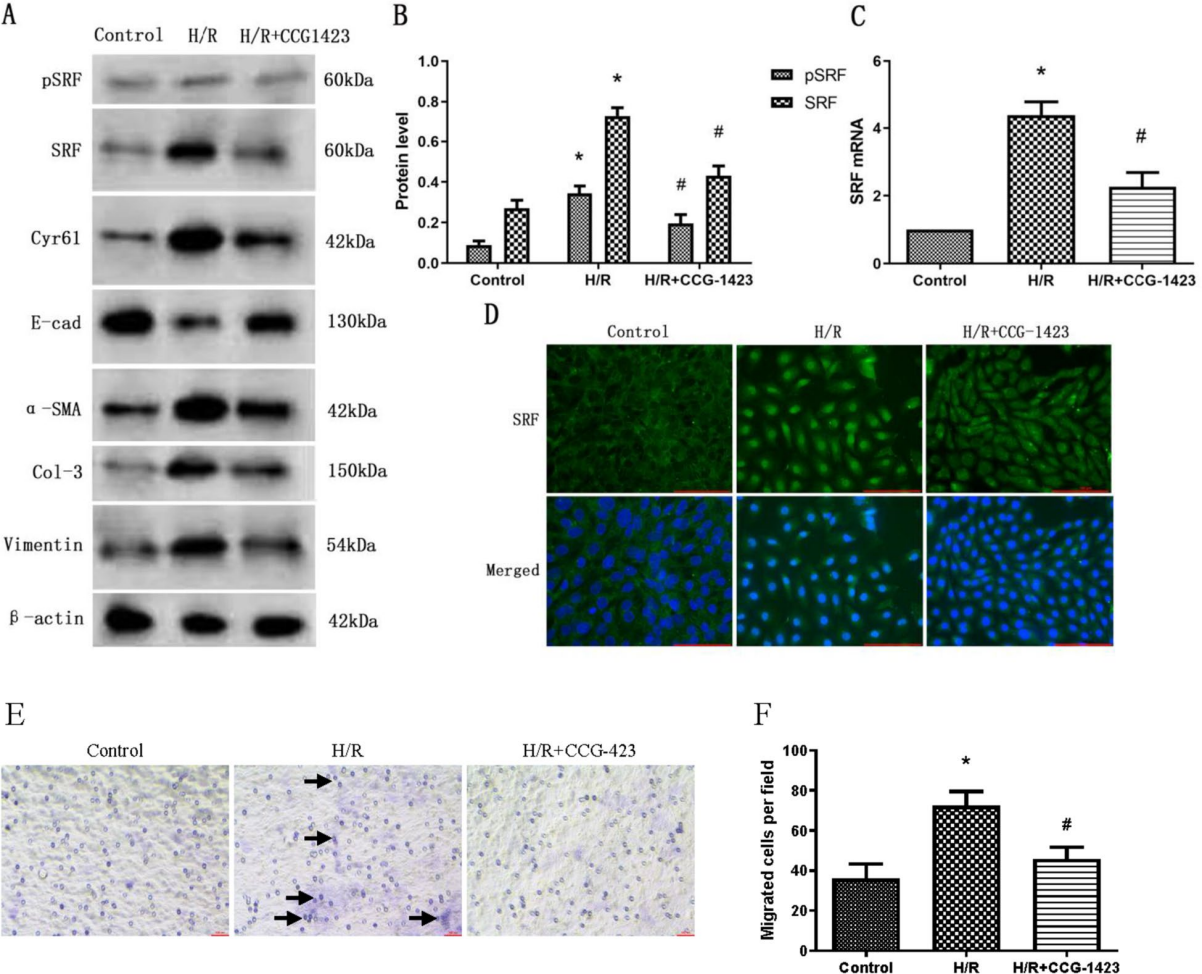
Suppression of SRF preserved the phenotype of TCMK-1 cells after H/R treatment. TCMK-1 cells were pretreated with CCG-1423 (2 μM) or DMSO for 1 h, followed by hypoxia treatment for 12 h followed by reoxygenation treatment for 24 h. **A** Protein expression of pSRF, SRF, E-cadherin, collagen-3, α-SMA and vimentin measured by western blot analysis. **B** Quantitative determination of the pSRF and SRF protein levels. **C** Quantitative RT–PCR analysis of SRF mRNA expression. **D** Immunofluorescence staining for SRF at an original magnification of 400 ×. Bar = 100 μm. **E** Representative micrographs of the transwell chamber migration assay at an original magnification of 200 ×. Arrowheads indicate some of the migrated cells. Bar = 50 μm. **F** Quantitative analysis of the number of migrated TCMK-1 cells per field in three groups. Data are representative of at least three independent experiments. *P < 0.05 versus the control group; ^#^*P* < 0.05 versus the H/R group

To assess whether curtailing SRF could offer protection to TCMK-1 cells against H/R-induced injury, we employed CCG-1423, a small molecular inhibitor of SRF. The modus operandi, specificity, and potency of CCG-1423's capacity to inhibit SRF had been previously well-established [27] Following a 24-h period of H/R treatment in TCMK-1 cells, CCG-1423 led to a reduction in both SRF and pSRF levels (Fig. 5A–C). As depicted in Fig. 5D, CCG-1423 obstructed the expression and translocation of SRF in TCMK-1 cells provoked by H/R. Furthermore, CCG-1423 notably impeded the elevation of collagen-3, vimentin, and α-SMA while also mitigating the reduction in E-cadherin. Furthermore, the inhibition of SRF notably decreased the migratory capability of TCMK-1 cells across transwell filter membranes (Fig. 5E, F), indicating that the reduction of EMyT solely through inhibited SRF was adequate to reduce the migratory potential of TCMK-1 cells. These effects collectively worked to prevent the TCMK-1 cells from undergoing EMyT (Fig. 5A).

### Inhibition of SRF suppressed the Cyr61 expression in vitro

**Fig. 6:**
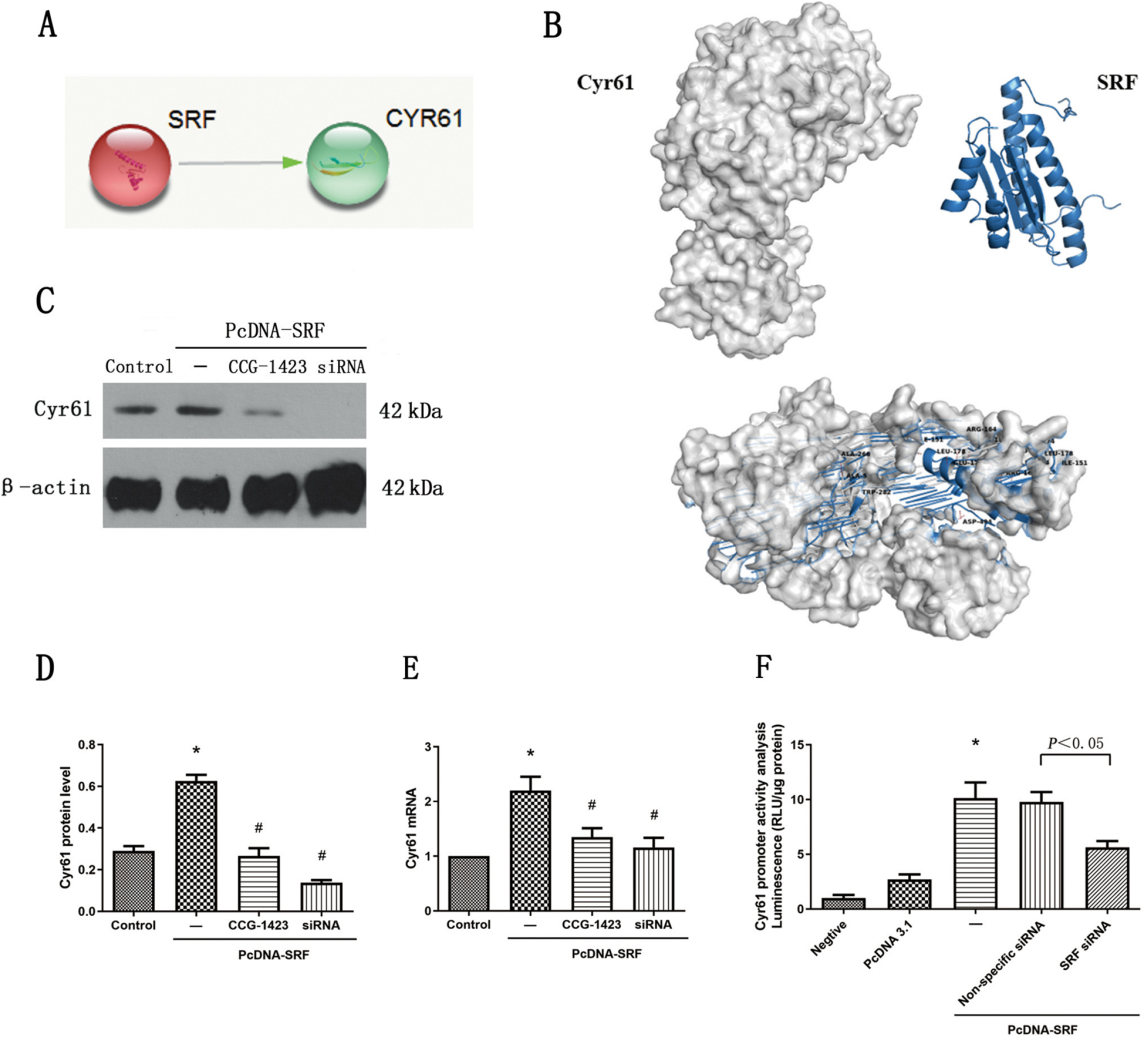
Inhibition of SRF suppressed the upregulation of Cyr61 expression in vitro. **A** STRING search result from website (https://string-db.org/). **B** Molecular docking showed that SRF could bind tightly to Cyr61. **C–E** TCMK-1 cells were transfected with pcDNA3.1–SRF or empty pcDNA3.1 vectors. After 24 h, the cells were incubated with CCG-1423 or SRF siRNA for another 24 h. The protein and mRNA expression levels of Cyr61 were measured by western blot analysis and quantitative RT–PCR. (F) Cyr61 promoter activity was analyzed by measuring luminescence. Data are representative of at least three independent experiments. *P < 0.05 versus the control group; ^#^*P* < 0.05 versus the pcDNA–SRF group

To study the potential connection between SRF and Cyr61, we conducted a search on the STRING database (https://string-db.org/) and identified indications that SRF might exert a promotive influence on Cyr61 (Fig. 6A). In addition, molecular docking analyses demonstrated a potential close interaction between SRF and Cyr61, indicating the likelihood of their interplay. For further validation of the relationship between them, an experimental approach combining SRF overexpression and inhibition was performed. As illustrated in Fig. 6C–F, both CCG-1423 treatment and SRF-specific siRNA markedly curtailed the Cyr61 expression prompted by SRF overexpression. This observation suggests that suppression of SRF through CCG-1423 or SRF siRNA could effectively negate a crucial transcription factor that governs EMyT under various conditions. To establish a more direct role of SRF in regulating Cyr61, a luciferase assay was employed. As described in Fig. 6F, the elevation in Cyr61 promoter activity resulting from SRF overexpression was substantial, while such activity was notably diminished when SRF siRNA was introduced. These outcomes aligned with the effects seen after CCG-1423 treatment, further corroborating the notion of SRF's direct influence on Cyr61.

### Suppression of SRF attenuate kidney injury

**Fig. 7:**
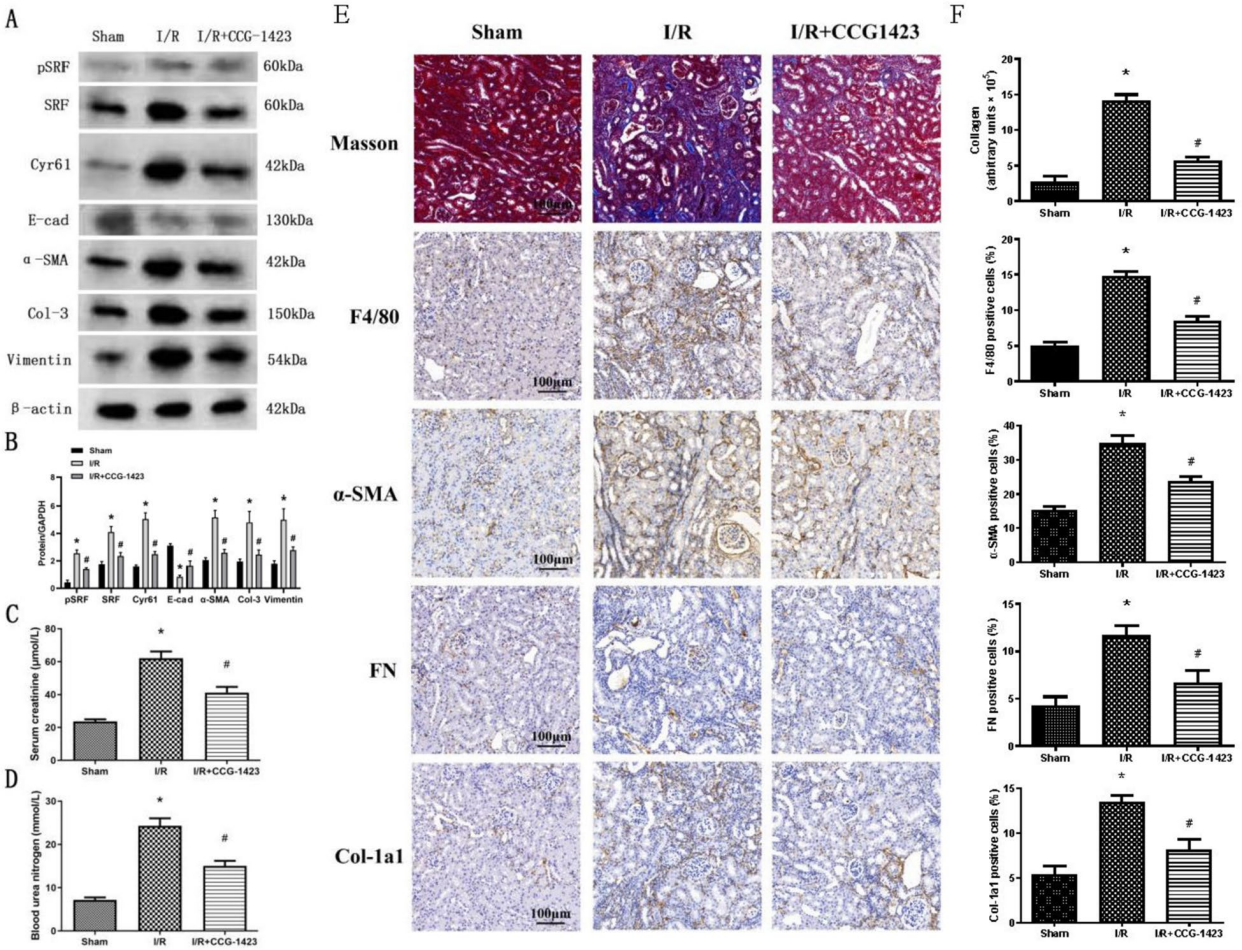
Suppression of SRF attenuated renal failure and renal tubulointerstitial fibrosis in rats. **A, B** Protein expression levels of pSRF, SRF, Cyr61, E-cadherin, α-SMA, collagen-3 and vimentin measured by western blot and quantitative analyses. **C, D** Reduction of Scr and BUN in I/R rats under CCG-1423 treatment. **E** Renal tubulointerstitial fibrosis in the three groups measured by Masson staining. Immunohistochemical staining for F4/80, α-SMA, FN and collagen-1a1 in the three groups. **F** Quantification of collagen, F4/80, α-SMA, FN and collagen-1a1 was calculated using Image J analysis software. Bar = 100 μm. N = 6 in each group. *P < 0.05 versus the sham group; ^#^*P* < 0.05 versus the I/R group

As shown in Fig. 7A, B and E, F, the administration of CCG-1423 to suppress SRF exhibited a marked impediment on the elevation of collagen-3, vimentin, α-SMA, F4/80, α-SMA, FN, collagen-1a1, SRF, and Cyr61 levels while simultaneously counteracting the decrease in E-cadherin expression within renal medulla tissue samples. Masson revealed the presence of renal tubulointerstitial fibrosis in the I/R group, with notable reduction observed in tubulointerstitial fibrosis following a 4-week CCG-1423 treatment in the I/R + CCG1423 group in comparison with the I/R group (Fig. 7E, F). Furthermore, CCG-1423 led to an improvement in renal function (as indicated by Scr and BUN levels) in I/R rats (Fig. 7C, D). In contrast to the vehicle control, the administration of CCG-1423 at a dose of 0.02 mg/kg of body weight led to a significant approximately 35% reduction in both Scr and BUN levels. These findings collectively highlight that targeting SRF through the application of a small molecular suppressor effectively blocked TEC EMyT, renal tubulointerstitial fibrosis, and renal failure subsequent to AKI.

## Discussion

Studies have shown that SRF is essential for the maintenance of RTEC morphology by regulating the actin cytoskeleton [28].What is more, a recent study established a vital role for SRF in the maintenance of podocyte structure and function, indicating a tight relation between kidney injury and SRF [29]. Remarkably, SRF were increased and activated in ischemic AKI both in vivo (Figs. 1, 2 and 7) and in vitro (Fig. 3). SRF expression was upregulated in renal medulla tissue samples from the I/R rats (Fig. 7), which was characterized by dramatic renal tubulointerstitial fibrosis. This is consistent with an our previous study, which shows that SRF is remarkably upregulated in AKI and can be a potential new early diagnostic biomarker of AKI [30].

It has been shown that SRF regulates EMyT in a variety of renal injuries (high glucose, high uric acid, obstruction), leading to renal fibrosis [13, 20, 31–33]. Moreover, EMyT in RTEC is an early characteristic change of renal tubulointerstitial fibrosis after renal injury. After the occurrence of AKI, RTEC can restore the normal structure of the kidney by repairing the damaged cells, while the sustained injury stimulates RTEC to develop EMyT [34]. Therefore, SRF overexpression in TCMK-1 cells upregulated a lot of myofibroblast markers and inhibited a lot of epithelial markers as well (Fig. 4). Those changes of cytoskeletal factors (α-SMA) and cell–cell adhesion factors (E-cadherin) can lead to EMyT and renal failure. Interestingly, SRF overexpression increased TCMK-1 cell motility and migration, which may be a result or a portion of EMyT, resulting in functional nephron numbers decrease. To sum up, those findings indicated that the increased SRF expression is competent to alter TEC phenotypes and mobility that are related to EMyT.

Although the specific pathogenesis by which SRF promotes EMyT of TECs remains to be demonstrated, these mechanisms appear to be related to the ability to induce the expression of Cyr61. Cyr61 is a secreted protein rich in cysteine, which has heparin-binding activity and can play different physiological functions by binding to integrin ligands and participating in various signaling pathways [35]. Studies have shown that Cyr61 plays a role in regulating RTEC apoptosis, fibroblast activation, proliferation and senescence, and interstitial angiogenesis in early IR–AKI[36, 37], which can protects the tubular epithelium. However, in the recovery stage of I/R–AKI, Cyr61 can promote fibrosis [38], and blocking Cyt61 can reduce renal inflammation and fibrosis after I/R–AKI [39]. Cyr61 also promotes fibrosis after lung injury by activating the TGF–β-smad3 signaling pathway in fibroblasts [40]. As early as 1991, Branko V. Latinkic et al. [41] found that SRF can exert a lasting promoting effect on the transcription of Cyr61 by binding to the promoter of the Cyr61 gene. Our results show that forced expression of SRF dramatically increased Cyr61 level (Fig. 6). Suppressing SRF through CCG-1423 or SRF siRNA inhibited H/R-mediated TEC EMyT, Cyr61 induction and renal failure. In summary, these findings indicate that SRF may play an important role through Cyr61 in the chronic progression after AKI.

CCG-1423 was reported to be a hopeful small molecular substances to prevent the progression of RCC [17]. The present research showed SRF might also be an attractive treatment target point in the chronic progression of AKI. In I/R rats, CCG-1423 ameliorated EMyT, renal failure and renal tubulointerstitial fibrosis by a dose-dependent manner (Fig. 7), which is in line with the role of SRF in vivo (Fig. 5). No drugs have been shown to treat the chronic progression of AKI by inhibiting EMyT. However, this study demonstrates that CCG-1423 may be an attractive pharmacological compound for improving the chronic progression of AKI through blocking EMyT, which may fill a gap in the AKI–CKD field.

It should be underlined that the present study has several limitations and defects, because we only used an I/R rat model and an immortalized TCMK-1 cell line. Whether these data can be extended to cultured primary TECs or other types of AKI remains to be illuminated, and also future studies should employ subcellular fractionation to precisely delineate the dynamics of SRF activation and nuclear translocation. Moreover, whether our results can be expanded to AKI of human remains to be addressed. Last but not least, this study suggests that SRF plays a role in the chronic progression after AKI, but the role of SRF in the acute phase of AKI needs further study.

## Conclusion

In summary, this study has demonstrated that SRF/Cyr61 pathway is upregulated in ischemic AKI, which may play a critical part in promoting dysfunction and EMyT of RTECs. Therefore, our findings demonstrate proof of the principle that the pharmacological compound targeting the SRF can be a promising strategy in the treatment of the chronic progression of AKI.

## Data Availability

Data will be made available upon request from the corresponding author.
